# Evaluation of the mechanical properties and clinical application of nickel–titanium shape memory alloy anal fistula clip

**DOI:** 10.3389/fsurg.2023.1235666

**Published:** 2023-08-23

**Authors:** Heng Deng, Ming Li, Xiaoli Fang, Jun Zhang, Jianmin Wang, Kun Tang, Ran Tang, Ru Jia, Ying Han, Yang Shi, Yu'ang Dong

**Affiliations:** ^1^ Department of Anorectal Surgery, Second Affiliated Hospital of Anhui University of Chinese Medicine, Hefei, China; ^2^ Department of Anorectal Surgery, First Affiliated Hospital of Anhui University of Traditional Chinese Medicine, Hefei, China; ^3^ College of Traditional Chinese Medicine, Anhui University of Chinese Medicine, Hefei, China

**Keywords:** anal fistula clip, internal orifice, mechanical properties, nickel–titanium alloy, clinical application

## Abstract

**Objective:**

The study investigates the mechanical properties of a nickel–titanium shape memory alloy anal fistula clip (NiTi-AFC), studies the surgical method of treating anal fistula, and evaluates its clinical efficacy.

**Methods:**

The anal fistula clip was formed in nickel–titanium alloy with a titanium content of 50.0%–51.8%. The mechanical properties and chemical properties were tested. A total of 31 patients with anal fistula were enrolled between 1 January 2020 and 1 January 2023. All patients underwent internal orifice closure surgery using NiTi-AFC, and anorectal magnetic resonance or ultrasound was performed before surgery and 6 months after surgery for diagnosis and evaluation. Fistula cure rates, length of stay, perianal pain, and Wexner incontinence scores were retrospectively compared between patients treated with NiTi-AFC and patients treated with other surgical methods.

**Result:**

NiTi-AFC has a density of 6.44–6.50 g·cm^−3^, with a shape-restoring force of 63.8 N. The corrosion rate of NiTi-AFC in 0.05% hydrochloric acid solution at atmospheric pressure and 20°C is approximately 6.8 × 10^−5^ g·(m·h)^−1^. A total of 31 patients (male/female: 19/12, age: 43.7 ± 17.8 years) were included. Among them, 22.6% (7) had multiple anal fistula, 16.1% (5) had high anal fistula, and 48.3% (15) had perianal fistula Crohn's disease. In total, 12.9% (4/31) did not achieve primary healing, underwent fistula resection, and eventually recovered. A retrospective analysis showed that the fistula healing rate, length of stay, and anal pain of NiTi-AFC treatment were similar to those of other traditional surgeries, but the Wexner incontinence score was significantly lower.

**Conclusion:**

NiTi-AFC has shape memory properties, corrosion resistance, superelastic effect, and surface cell adhesion. It is applied to internal orifice closure surgery of anal fistula, with good therapeutic effect, and can protect the anal function.

## 1. Introduction

Nitinol is probably the most active new functional material in the world today (1). Alloy has been widely used in aerospace, aviation, machinery, instrumentation, and petrochemical industry because of its extraordinary shape memory effect and hyperelastic effect (2). It is also used in bed implantation as a biomedical engineering material (3). Nickel–titanium shape memory alloys have had some success in reconstructive surgery (4). However, there are still some limitations in the clinical application of NiTi alloy because of the different surgical sites and specific requirements for alloy materials (5).

The anal fistula is the duct between the perianal skin and the rectoanal canal, resulting from chronic infection and epithelialization of the drainage duct (6). Its pathological elements include the primary internal orifice, fistula, and secondary external orifice (7). These abnormal pathological structures determine the need for surgical treatment of anal fistulas (8). The principle of operation is to clear the internal orifice, eliminate the fistula, smooth the drainage, reduce sphincter injury as much as possible, and protect anal function (9). According to the treatment principle of anal fistula and the characteristics of nitinol alloy, we tried to design an anal fistula clip to meet the needs of clinical treatment. This study will clarify the clinical application of combining the excellent properties of nitinol with the principles of treatment of anal fistula.

## 2. Materials and methods

### 2.1. NiTi shape memory alloy anal fistula clip design

**Figure 1:**
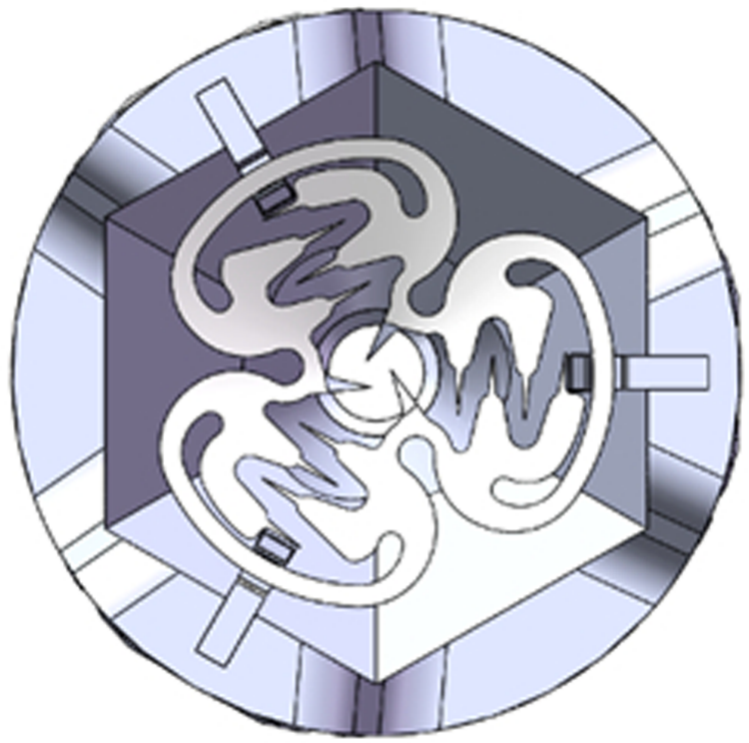
Diagram of NiTi shape memory alloy anal fistula clip. Original shape: three identical-sized alloy blades on the same plane.

According to the shape and measurement results of the anal fistula, to solve the problem of the opening and infection of the internal orifice, NiTi-AFC designed a 0.5 mm thick round nickel–titanium shape memory alloy clip (with an open diameter of 14 mm). The three fan-shaped alloy blades form a centripetal closed design structure with a unidirectional memory design (Figure 1). The alloy blade is perpendicular to the ring plane at the deformation temperature (0 – 5°C), and the recovery temperature is 34 – 41°C.

**Figure 2:**
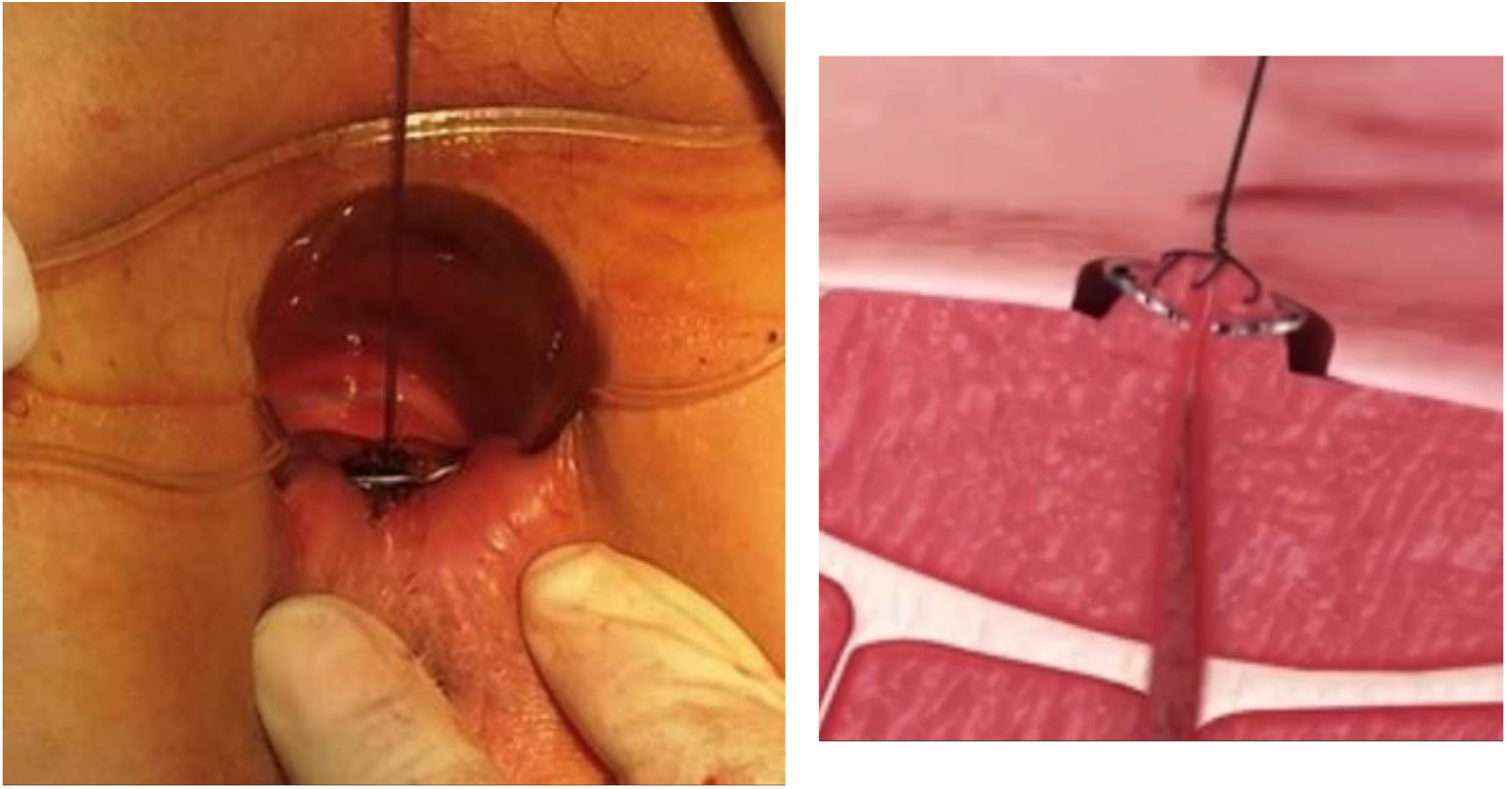
Diagram of NiTi-AFC with the closed internal orifice. When the temperature was close to body temperature, the three alloy blades returned to their original shape and formed a combined force to close the internal orifice of the fistula.

The alloy composition by mass is Ti (50% – 51.8%), Ni. NiTi-AFC has a density of 6.44–6.50 g·cm^−3^. Refrigerated NiTi memory alloy retains its shape easily, even after deformation; the three blades are stretched around the internal orifice. When the temperature returns to the body temperature, centripetal forces converge the internal orifice and surrounding tissues into one (Figure 2).

### 2.2. Mechanical property test

The tensile strength and superelastic limit were measured at 37°C. A constant temperature water bath box (Model: AHE-100, Chengdu Tai Meng, China) was used to control the experimental temperature with a temperature deviation of 0.1°C. At 0 – 5°C, the sample was deformed, and the blade opened upward. The deformed sample was placed in a constant temperature water bath, and the temperature was gradually increased until the product returned to its original shape. At this point, the temperature was the phase transition temperature of NiTi-AFC, and the maximum value on the Radial Force Tester (YOLO-YFG800, Shanghai Yaolin, China) is the shape-restoring force.

### 2.3. Chemical property test

At 20°C and normal atmospheric pressure, the NiTi shape memory alloy anal fistula clip (NiTi-AFC) was placed into 0.05% hydrochloric acid solution for 96 h for the corrosion test.

### 2.4. Clinical evaluation methods

With the approval of the Ethics Committee (approval number: 2018AH-25), 31 patients with anal fistula admitted between 1 January 2020 and 1 January 2023 were included and underwent internal orifice closure surgery with NiTi-AFC. Perianal magnetic resonance imaging (Philips Ingenia 1.5-T scanner, Philips Medical Systems Nederland B.V.) was performed to evaluate the fistula course and type before surgery and the healing status at 6 months after surgery. An anorectal color ultrasound (ESAOTE Mylab90) was performed instead of perianal magnetic resonance imaging if the patient could not undergo the procedure. The pain score on the first, third, and seventh days after surgery was based on the pain number scoring method (10). The length of stay is counted by day. At 6 months after surgery, the rate of primary fistula healing was calculated based on clinical symptoms combined with magnetic resonance imaging (or anorectal duplex color ultrasound), and anal incontinence was assessed based on the Wexner incontinence score (11). These observation indexes were retrospectively compared in 31 patients who underwent conventional operations.

### 2.5. NiTi-AFC internal orifice closure surgery

Following subarachnoid anesthesia, an alloy probe was used to examine the internal orifice of the anal fistula from the external orifice, and the 1 cm mucous tissue around the internal orifice was removed. The fibrotic tissue inside the fistulas was removed using a metal brush, and the external orifice of the anal fistula was enlarged for drainage. The internal orifice and surrounding tissues were lifted, and NiTi-AFC at 0–5°C was placed around it. After rewarming, the internal orifice was clamped. NiTi-AFC is often localized near the dentate line because most of the internal orifice opens at the anal recess, whereas the internal orifice of perianal fistula Crohn's disease (PFCD) often diverges from this point. NiTi-AFC was only placed when Crohn's disease was in remission, using biologics according to guidelines. A corresponding number of clips were placed according to the number of the internal orifices, and if multiple fistulas belonged to the same internal orifice, only one clip was placed. The NiTi-AFC was naturally detached or removed 2 weeks after surgery (12, 13).

### 2.6. Statistical methods

Mean ± standard deviation(s) were used to express data, which were analyzed using the SPSS 19.0 statistical software package. The scoring data were analyzed using Welch’s *t*-test, and the counting data were analyzed using the Wilcoxon rank sum test. A *P*-value of <0.05 was considered statistically significant.

## 3. Result

### 3.1. Mechanical properties

NiTi-AFC has a density of 6.44–6.50 g·cm^−3^, with a shape-restoring force of 63.8 N. The corrosion rate of NiTi-AFC in 0.05% hydrochloric acid solution at atmospheric pressure and 20°C is approximately 6.8 × 10^−5^ g·(m·h)^−1^ (Table 1).

**Table 1:** Physical characteristics of NiTi shape memory alloy anal fistula clip.

Characteristic	Number	Unit
Melting point	1,240–1,310	°C
Density	6.44–6.50	g·cm^−3^
Thermal conductivity	0.034	W·(m·K)^−1^
Superelastic limit	12–16.4	%
Shape-restoring force	63.8	*N*
Corrosion rate	6.8 × 10^−5^	mm·m^−1^

### 3.2. Clinical application results

**Figure 3:**
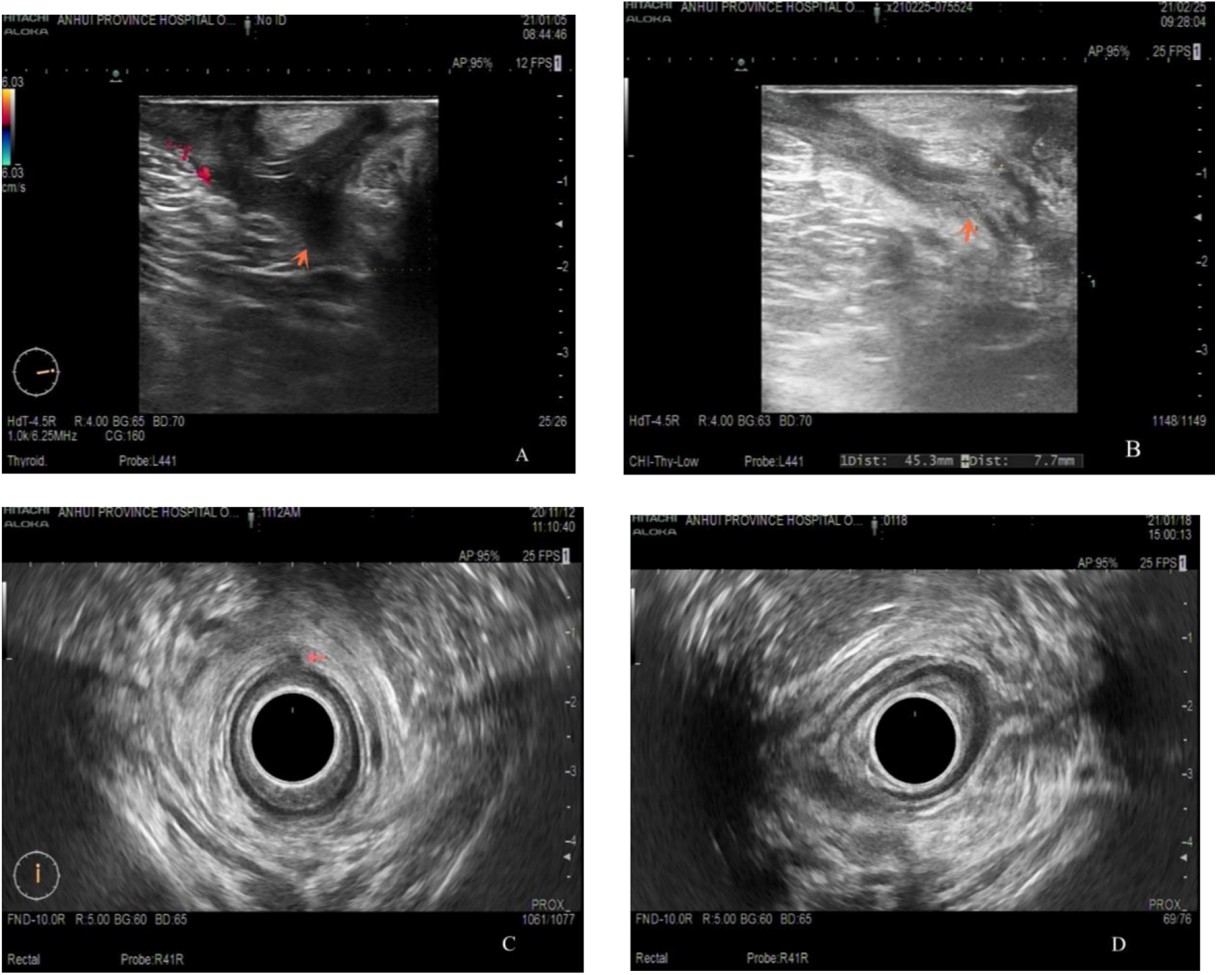
Dual-plane color ultrasound images of the anal canal of a young male before (**A,C**) and after (**B,D**) receiving NiTi-AFC internal orifice closure. The orange arrow indicates that the low-echo fistula has been replaced by a high-echo scar.

**Figure 4:**
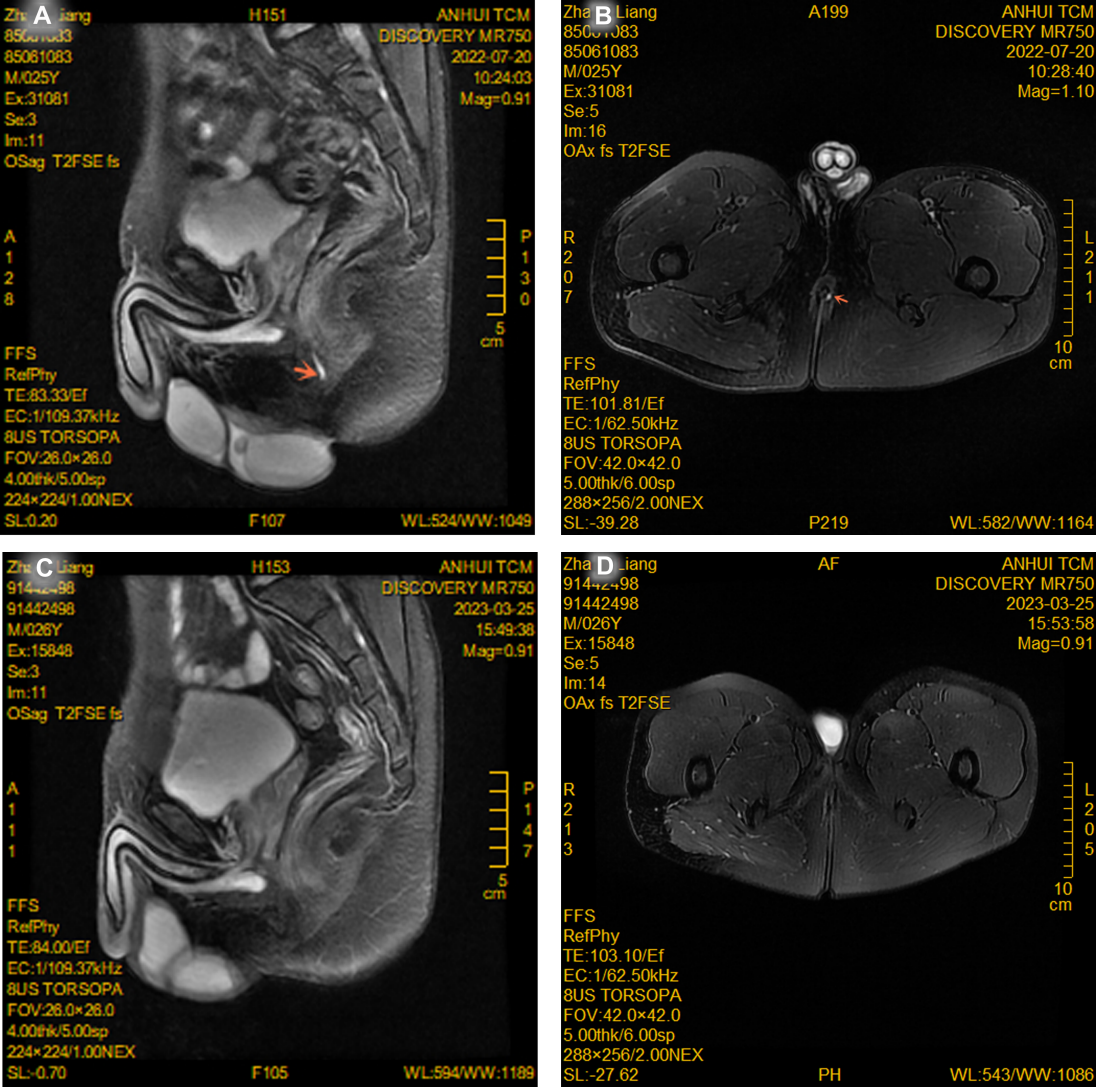
Magnetic resonance imaging of a young male before (**A,B**) and after (**C,D**) receiving NiTi-AFC internal orifice closure. The T2 phase showed that the fistula shadow with a high signal tended to disappear (red arrow point).

NiTi-AFC was administered to 31 patients with anal fistula (male/female: 19/12, age: 43.7 ± 17.8 years). Among them, 22.6% (7) had multiple anal fistulas, 16.1% (5) had high anal fistula, and 48.3% (15) had PFCD, which were specially included after using biological agents and achieving mucosal healing. The follow-up period ranged from 1 to 6 months, and the primary healing rate of fistulas within 6 months after surgery was calculated. For all fistulas to heal, anorectal color ultrasound was required to show that the low-echo fistulas became hyperechoic scar tissues or that the high-signal fistulas disappeared in stage T2 magnetic resonance imaging (Figures 3, 4). Four patients (three PFCDs) did not heal in one stage within 6 months and eventually healed after fistulotomy.

A total of 31 patients with anal fistula (male/female: 18/13, age: 44.7 ± 16.1 years, 19.4% (6) had multiple anal fistulae, and 19.4% (6) had high anal fistula) who had recently received other surgical methods (rectal mucosal muscle flap propulsion repair, anal fistula resection, anatomy including intersphincteric fistula ligation, and transanal opening of intersphincteric space surgery) in the anorectal center were selected as the control group for retrospective analysis. The postoperative fistula cure rate, length of hospital stay, perianal pain, and Wexner urinary incontinence score were compared between the two groups (Table 2).

**Table 2:** Fistula healing rates, length of hospital stay, perianal pain, and the Wexner incontinence scores were compared between the two groups.

Patient group	*n*	Perianal pain scores	Wexner incontinence scores	Fistula healing rates	Days of hospital stay
Control	31	3.52 ± 0.65	4.27 ± 3.43	89.2%	8.75 ± 2.47
NiTi-AFC	31	3.59 ± 0.81	3.09 ± 2.39	87.1%	8.97 ± 1.42
*P*-value		0.188	<0.001	0.256	0.397

## 4. Discussion

Anal fistula patients accounted for 3.5% of the patients who visited the anorectal department (14). Due to the large population base, the total number of patients remains high. The pathological structure of the anal fistula and the physiological function of the anus require surgical treatment and protection (15). Anorectal specialists have made a great deal of material practice in this field. To avoid incontinence caused by anal fistula surgery, cotton thread was used as a medical material in China's Ming Dynasty. The cotton thread in anal fistula surgery gradually severs the external anal sphincter under the action of gravity. Rubber bands have recently been used in anal fistula suture surgery to prevent incontinence by slowly cutting the anal sphincter with centripetal elasticity (16). With the development of science and technology, some emerging materials have also been used for fistula filling to treat anal fistulas, such as allogenic dermal acellular matrix (17) and mesenchymal stem cells (18).

In the early 1970s, the medical basic application research of the alloy material has been carried out, focusing on the histological observation of the alloy material after implantation in animals, and it has been applied in orthodontics (19). With additional research, nitinol was found to have distinctive properties such as unique memory of geometric shapes, superelasticity, high strength, resistance to rust, non-magnetic properties, wear resistance, and fatigue resistance (20). Due to its non-toxic resistance to human medium corrosion and good cell adhesion, nitinol is widely used in orthopedics (21), gastrointestinal and facial surgery (22, 23), cardiac surgery (24), etc.

Whether the mechanical and chemical properties of NiTi-AFC meet the requirements of anal fistula treatment is an important reference for its clinical application. The experimental test shows that NiTi-AFC can be fixed continuously at body temperature, and its active compression enhances the fixation effect. The shape-restoring force of 63.8 N is sufficient to close the internal opening of the anal fistula and its surrounding tissues. The corrosion resistance of NiTi-AFC is sufficient to cope with the environment of excrement in the rectum. The ring with a thickness of 0.5 mm and a diameter of 14 mm is consistent with the structure of the inner wall of the rectum. As a result, NiTi-AFC can mask the shortcomings of internal fixtures. Furthermore, in clinical application, NiTi-AFC internal orifice closure performed well in fistula healing rate, length of stay, postoperative anal pain, and other aspects. NiTi-AFC showed superior results compared with conventional surgery in terms of postoperative incontinence score. In addition, the procedure is simple and easy to master, the equipment is affordable, and the economic burden is low.

The over-the-scope clip (OTSC), alloyed with the NiTi-AFC clip, is used for leakage management after laparoscopic sleeve gastrectomy by clamping the rupture of the digestive tract wall (12). OTSC is performed under endoscopy, and NiTi-AFC is performed under direct vision. The OTSC has a symmetrical double-blade structure, while the NiTi-AFC clamp has a three-blade structure, with more continuous centripetal force.

However, as a foreign body, NiTi-AFC is associated with an increased risk of allergic reactions (25). Due to the lack of PFCD patients in the control group, the comparison group is not defined more precisely. These characteristics need more clinical samples and randomized controlled trials.

## 5. Conclusion

The design of the nitinol material anal fistula clip conforms to the characteristics of the anatomical structure of the anal fistula. The fixed force can meet the mechanical requirements of internal orifice closure. NiTi-AFC is simple and practical in treating anal fistula, with good clinical effect and protection of the sphincter.

## Data Availability

The datasets presented in this study can be found in online repositories. The names of the repository/repositories and accession number(s) can be found below: Some raw data images are available at https://www.jianguoyun.com/p/DSfY1BkQ19jVCxjU24oFIAA.
